# When a glabellar dermoid cyst is not the whole story: the significance of MRI in excluding intracranial segments

**DOI:** 10.1186/s12887-026-08016-6

**Published:** 2026-11-06

**Authors:** Rebekka Wiedenhoefer, Ursula Feige, Mukesch Shah, Lennart Gunst, Tobias Feuchtinger, Alexander Puzik

**Affiliations:** 1 Department of Pediatric Hematology, Oncology and Stem Cell Transplantation, Medical Center, Faculty of Medicine, Children’s Hospital, University of Freiburg, Freiburg, Germany; 2 Department of Neuroradiology, University Medical Center Freiburg, University of Freiburg, Freiburg, Germany; 3 Department of Neurosurgery, University Medical Center Freiburg, University of Freiburg, Freiburg, Germany; 4 Department of Pediatrics, Adolescent Medicine and Neonatology, Faculty of Medicine, University Children’s Hospital, Freiburg, Germany

**Keywords:** Dermoid cyst, Epidermoid cyst, Intracranial extension, MRI, Case report

## Abstract

**Background:**

Epidermoid and dermoid cysts are one of the most common benign pediatric tumors of the skull. Ultrasonography is usually the diagnostic tool of choice for their evaluation and diagnosis. Although benign, local growth with cosmetic issues, bone erosion, and occasional intracranial involvement generally warrant surgical resection. Since epidermoid and dermoid cysts may be seen by a wide range of medical disciplines, standardized recommendations for optimal diagnosis and treatment are necessary.

**Case presentation:**

We report the case of a 14-month-old boy, who underwent neurosurgical removal of a glabellar dermoid cyst classified as an extracranial lesion, and in whom an intracranial lesion was identified on follow-up MRI, represents a rare and unusual clinical course. Ten months after surgery, a surveillance MRI revealed an intracranial lesion in the right frontal parasagittal region, while the child had developed no symptoms. Very careful retrospective review of the initial MRI suggested that an extremely small intracranial part of the lesion might have been faintly visible upon knowledge of the further clinical course. The intracranial lesion was subsequently resected and diagnosed as an epidermoid cyst. In our mini-review, we have compiled evidence that intracranial extension is more common than expected and particularly occurs in craniofacial midline lesions but is definitely not restricted to them.

**Conclusion:**

Together, this case and literature review highlight the importance of careful preoperative assessment and interdisciplinary discussion to adequately determine the extent of craniofacial dermoid and epidermoid cysts before surgery. This case illustrates that a subtle intracranial lesion may not be recognizable on initial image interpretation despite MRI having been performed. Imaging follow-up is definitely recommended when intraoperative findings raise concern about deeper extension.

## Background

Epidermoid and dermoid cysts are one of the most common pediatric tumors of the skull and scalp, with an incidence of 16–33% of all skull lesions [1–3]. Both lesions arise from misfolded ectoderm [4]. They often present as indolent subcutaneous masses around the orbit but may also involve the periosteum and bone and can occasionally extend intracranially [5]. Nevertheless, they present a challenge for optimal diagnostic and therapeutic management carried out at different medical disciplines and specialization levels of medical care. Although benign, complete surgical excision of the lesion is the therapy of choice and indicated for most epidermoid and dermoid cysts, as the cysts may grow larger or rupture as they progress and then cause cosmetic issues, inflammation, bone erosion or neurologic deficits when localized intracranially [6, 7]. Malignant transformation of the cysts rarely occurs, most commonly into squamous cell carcinoma, typically in adulthood (average age 54 years), and most often arising from intracranial dermoid or epidermoid cysts [8]. Recurrences after surgical care are very rare [5]. While surgery remains the standard treatment, especially for lesions located in the midline structures, management should be individualized according to the lesion location and suspected extension [5, 9].

To assess subcutaneous lesions in children, an ultrasound examination is usually performed first. Epidermoid and dermoid cysts appear as well-circumscribed, round to oval, sometimes lobulated, avascular lesions. Inside, there are echogenic lesions caused by debris of compacted keratinized squamous epithelium [10]. Magnetic resonance imaging (MRI) was considered to be necessary if the cysts occur along the midline of the scalp, as intracranial spread is reported here [11]. On MRI, epidermoid cysts, consisting of keratin lamellae, appear isointense to the cerebrospinal fluid, whereas dermoid cysts are hyperintense in T1-weighted images due to additional skin appendages in the cyst wall that secrete an oily fluid into the cyst cavity [12]. The sensitivity and specificity of MRI for detecting intracranial spread of the lesion was reported to be 100% and 95.7%, respectively, whereas the ultrasound only had a sensitivity of 50% [13].

In this context, we report a case in which an intracranial lesion was identified on surveillance MRI after removal of a glabellar dermoid cyst that had initially been classified as extracranial. Upon knowledge of the further clinical course, retrospective review suggested that the intracranial lesion might already have been faintly visible on the initial MRI. This case highlights the importance of careful and interdisciplinary preoperative assessment and the role of MRI in assessing these lesions.

## Case presentation

**Fig. 1:**
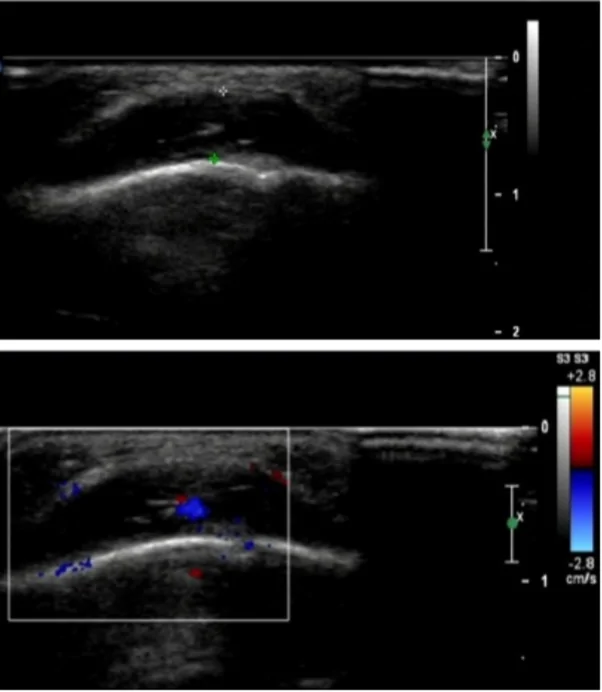
Initial ultrasound of the cyst showing an encapsulated, homogeneous and poorly vascularized mass

A 14-month-old boy presented with an increasing mass between the eyebrows. The lesion, which had been present since birth, repeatedly discharged white secretions. Clinical examination revealed a 3–4 cm protrusion of the glabellar region. Laboratory work demonstrated no abnormalities. Ultrasound revealed an encapsulated, homogeneous and poorly vascularized mass (Fig. 1).

**Fig. 2:**
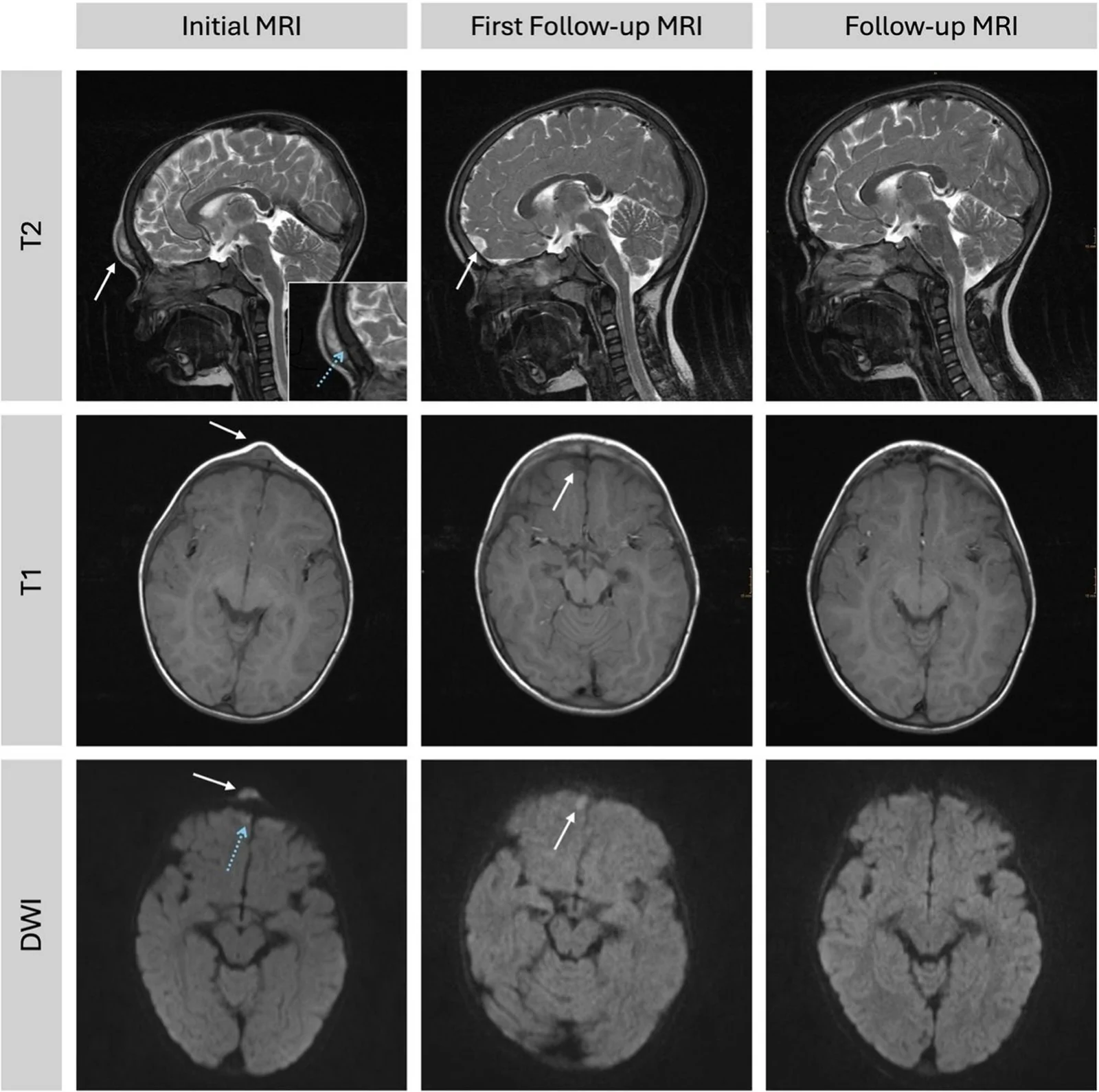
MRI findings at three representative time points. Left: Initial MRI. Middle: Follow-up MRI, approximately 10 months after the initial examination, showing the intracranial lesion. Right: Follow-up MRI, approximately 4 months after the second surgery. Images are shown from top to bottom as sagittal T2-weighted, axial T1-weighted, and axial diffusion-weighted imaging (DWI). White arrows indicate the lesions identified on the respective examinations. Blue dotted arrows on the initial MRI indicate subtle findings that, on retrospective review, may suggest intracranial extension

MRI was performed according to our institutional policy. A 14-mm polylobulated extracranial mass with central T2 hyperintensity and peripheral T2 hypointensity was detected, indicating a suspected diagnosis of an epidermoid or dermoid (Fig. 2). The initial MRI was performed without contrast and included T1-weighted, T2-weighted and FLAIR sequences, as well as diffusion-weighted imaging and ADC maps. No intracranial connection or lesion could be identified in the initial neuroradiological assessment.

The case was discussed in our interdisciplinary tumor board, and the whole extracranial mass was removed by neurosurgery two weeks later. Intraoperatively, the mass was found to be attached to the underlying bone. A central indentation of the bone contained connective tissue strands extending into the mass. These strands were coagulated and divided, and the abnormal tissue at the bony attachment was burred away, leaving a small indentation in the bone. A small midline skin opening, considered to represent a dermal sinus, was also completely excised. The dura was not exposed or inspected. Histopathology confirmed a dermoid cyst, with keratinizing stratified squamous epithelium and skin appendages, including hair follicles and glandular structures.

Follow-up MRI was recommended three months after surgery because of the intraoperative findings. However, the family attended the follow-up examination only approximately ten months after surgery. The follow-up MRI included T1-weighted, T2-weighted and FLAIR sequences, as well as diffusion-weighted imaging and ADC maps. The MRI showed no evidence of residual tissue at the glabellar surgical site. However, it revealed an intracranial right frontal parasagittal lesion with diffusion restriction (Fig. 2). The child had developed no symptoms.

On retrospective review of the initial MRI, the intracranial lesion was considered to be extremely faintly discernible (see blue dotted arrows in Fig. 2). The subtle appearance of this finding explains why it was not described in the initial neuroradiological assessment. Therefore, it remained unclear whether the intracranial segment of the lesion was present initially and grew between the first surgery and surveillance MRI or resembled a recurrence.

After a preoperative contrast-enhanced MRI, the intracranial mass was again removed surgically via a craniotomy in the right frontal region. Intraoperatively, the frontal bone showed no abnormalities; following the craniotomy extending to the midline, the dura mater was opened and the tumor was completely removed. No connection between the lesion and the dura mater was found. Histopathological examination showed numerous keratin lamellae and small fragments of epithelium, without glandular components, leading to a diagnosis of an intracranial epidermoid cyst.

Postoperative MRI performed one day after surgery showed a resection cavity measuring up to 8 × 8 × 9 mm, with an adjacent area in which a small residual lesion could not be excluded. Follow-up MRI approximately four months after surgery (Fig. 2) showed no evidence of residual epidermoid. Subsequent examinations 14 and 25 months after surgery confirmed remission. The timeline of the case is presented in Table 1.

Informed consent was obtained from the patient’s legal guardian, and this report complies with the Declaration of Helsinki.

**Table 1:** Timeline

Patient’s age	Clinical Event	Findings and interventions
Since birth	Clinical presentation	Glabellar mass present since birth, with recurrent white secretions
14 months (mo)	Initial MRI	14-mm polylobulated extracranial glabellar lesion. No intracranial lesion identified.
	First surgery	Extracranial lesion excised. Fibrous strands extending into the lesion divided, and the underlying bony attachment burred. Histopathology confirmed a dermoid cyst.
2 years (y) and 1mo	Follow-up MRI	No residual tissue at the glabellar surgical site. Intracranial right frontal parasagittal lesion with diffusion restriction identified. No symptoms.
2y and 3mo		
	Second surgery	Intracranial lesion resected. Histopathology confirmed an epidermoid cyst.
	Postoperative MRI	Resection cavity measuring up to 8 × 8 × 9 mm; a small residual lesion could not be excluded.
2y and 8mo	Follow-up MRI	No evidence of residual epidermoid.
3y and 5mo	Follow-up MRI	No evidence of residual epidermoid.
4y and 6mo	Follow-up MRI	No evidence of residual epidermoid.

## Discussion

Although most dermoid and epidermoid cysts in childhood are located in subcutaneous tissue, intracranial involvement occurs in approximately 9–15% of reported cases [13, 14]. Since uncomplicated dermoid cysts might not be reported regularly, the unexpected high percentage of intracranial involvement might reflect a reporting bias, though. Intracranial extension occurs predominantly in craniofacial midline lesions, particularly in frontonasal dermoid cysts [13, 14]. However, intracranial extension has also been reported in other anatomical locations (Table 2), indicating that non-midline localization does not preclude intracranial involvement [3, 15]. We identified 129 pediatric cases with intracranial extension of a dermoid cyst or epidermoid cyst in a narrative mini-review of the literature, see Table 2 [6, 13, 14, 16–20].

**Table 2:** Reported cases of dermoid cysts with intracranial extension

Ref	Type	Author(s)	No. of Patients	Localization	Age	Imaging	Complications
[6]	CR	Barnard et al. (2012)	1	Frontotemporal	3y	CT	Edema, erosive bone changes of the sphenoid wing; findings consistent with osteomyelitis
[20]	CR	Yan et al. (2016)	1	Frontotemporal	21mo	MRI and CT	Fever at presentation; treated with amoxiclav and linezolid for 3 weeks
[16]	CR	Davis et al. (2020)	1	Nasal (Midline intranasal)	6mo	MRI	No complications after surgical removal
[17]	CR	Giugliano Villarroel et al. (2024)	1	Nasoethmoidal	14y	CT and MRI	No postoperative complications or recurrence
[18]	CR	Lietaer et al. (2025)	1	Nasofrontal	2y	MRI	None reported
[19]	OA	Overland et al. (2020)	38	Lateral brow (1), Midline nasal (10), Frontal (14), Occipital (7), Temporal (6)	Avg. 26mo (Stage IV), 44.3mo (Stage V)	Not specified per case; MRI/CT/ultrasound in general	Not applicable
[13]	OA	Meira Pazelli et al. (2024)	18	Nasal (5), Parieto-occipital (1), Frontotemporal (6), Forehead (6)	Not available	Not specified per case; MRI/CT/ultrasound in general	Not applicable
[14]	Review	Gierlotka and Bielecki (2026)	68	Lateral dermoids (eyebrow, orbital) (2); midline dermoids (66)	Range from 4y to 17y	CT or MRI	Not applicable

Abbreviations: CR = Case report; OA = Original article; CT = Computed tomography; MRI = Magnetic resonance imaging; mo = months; y = years

In addition to the potential growth of residual intracranial segments of extracranial lesions, patients with intracranial connections are at increased risk of developing meningitis, abscesses, or osteomyelitis if left untreated [6, 21]. Therefore, it is essential to determine the presence of an intracranial involvement of craniofacial dermoid or epidermoid cysts.

Since intracranial lesions cannot be reliably excluded by ultrasound [13], we routinely perform MRI in children with skull lesions when intracranial involvement is possible and discuss MRI findings in our interdisciplinary tumor board. In the present case, the initial MRI was performed under sedation with enterally administered chloral hydrate, which is regularly used in our institution. Nevertheless, sedation for pediatric MRI requires an individualized assessment of the need for sedation, potential risks, and available alternatives, with appropriate monitoring and adherence to established safety standards [15, 22].

Given the potentially serious consequences of an unrecognized intracranial connection, MRI should be considered in children presenting with dermoid or epidermoid cysts of the skull. Particular attention should be paid to craniofacial midline lesions, as these carry the highest reported risk of intracranial extension [14]. While contrast enhancement is not routinely required for dermoid and epidermoid cysts, it may be useful when atypical features raise concern for alternative diagnoses or when an intracranial connection is suspected. An interdisciplinary discussion of such cases can help ensure appropriate treatment.

This case illustrates the diagnostic challenge posed by an intracranial lesion identified on follow-up MRI after removal of a glabellar dermoid cyst. Although the lesion may have been subtly discernible on retrospective review of the initial MRI upon knowledge of the later clinical course, its presence at that time could not be established with certainty. Either the progression of an extremely small intracranial lesion or an intracranial recurrence (depending on the definition) are the most likely explanations for this rare clinical course. The case highlights the importance of careful review of preoperative imaging, extension of imaging to MRI and consideration of timely follow-up imaging when radiological or intraoperative findings raise concern about possible intracranial involvement.

The differing histopathological diagnoses of the intra- and extracranial lesions (dermoid and epidermoid cyst, respectively) may reflect the heterogeneous composition of a possible combined primary lesion. Both entities originate from ectodermal inclusions during embryonic development and may represent different manifestations of this underlying tissue. It is therefore conceivable that the initial lesion contained both dermoid and epidermoid components, which may have been unevenly distributed and consequently represented differently in the two specimens. Tissue fragmentation and sampling limitations may also contribute to the differing histopathological findings. Evolution of the histological characteristics of these lesions has also been described [4].

Since this case report represents a single case with a very rare clinical course, conclusions drawn from this case study must be confirmed by other case series and further evidence. The literature review was not conducted as a systematic review, which means that relevant publications on our topic may have been overlooked.

## Conclusion

Since intracranial extension of craniofacial dermoid or epidermoid cysts may have important implications for surgical planning, preoperative MRI and interdisciplinary discussion can help assess the extent of the lesion and identify possible intracranial involvement. In our patient, the intracranial lesion was identified on follow-up MRI, while being subtly discernible on retrospective review of the initial images upon knowledge of the clinical course, highlighting the importance of careful preoperative radiological assessment. Follow-up MRI 3–6 months after surgery should be considered when preoperative or intraoperative findings raise concern about possible deeper involvement or residual disease.

## Data Availability

No datasets were generated or analysed during the current study.
